# Psychological well-being in times of COVID-19: Associated factors and levels in the general population

**DOI:** 10.3389/fpubh.2022.860863

**Published:** 2022-10-03

**Authors:** Sara Ebling, Omid V. Ebrahimi, Sverre Urnes Johnson, Nora Skjerdingstad, Asle Hoffart

**Affiliations:** ^1^Department of Psychology, University of Bergen, Bergen, Norway; ^2^Department of Psychology, University of Oslo, Oslo, Norway; ^3^Modum Bad Psychiatric Hospital, Vikersund, Norway

**Keywords:** well-being, mental health, COVID-19, social distancing, metacognitions

## Abstract

The COVID-19 pandemic and living under social distancing restrictions have been hypothesized to impact well-being and mental health in the general population. This study investigated the general Norwegian adult population's well-being after implementing and lifting strict social distancing restrictions. The study was conducted through digital surveys; during the implementation of strict social distancing restrictions in March 2020 (T1) and 3 months later, when the preponderance of strict distancing restrictions was discontinued (T2). Well-being was measured at T2. Four thousand nine hundred twenty-one individuals participated, and a sensitivity analysis was conducted to ensure that the sample reflects the true Norwegian adult population. Hierarchical regression analyses show that contemporaneous employment status and positive metacognitions at T2 were associated with higher well-being. Negative metacognitions and the use of unhelpful coping strategies at T2 had a contemporaneous association with lower mental well-being. Negative metacognitions at T1 were associated with lower well-being scores, while positive metacognitions at T1 were positively associated with higher well-being. An indirect association between social distancing and lower well-being was found through heightened depressive symptoms. These results contribute to understanding how social distancing restrictions relate to general well-being, which may further contribute to designing proper strategies to strengthen mental health and well-being during challenging and unavoidable societal conditions.

## Introduction

After the outbreak of the COVID-19 pandemic, everyday life changed in profound matters among different populations worldwide. The World Health Organization (WHO) early published governmental guidelines for coping with virus transmission, including a comprehensive focus on reducing physical mobility (1). This included social distancing strategies such as the closure of schools and workplaces, adherence to a physical distancing of 1–2 meters between individuals, and quarantine or isolation of infected or high-risk individuals (2). As multiple social distancing strategies strode forward, more attention was brought to the potential adverse effect on mental health (3). A vast amount of literature has investigated how the psychopathology rates have increased in different populations during the COVID-19 pandemic and lockdowns, e.g., increased prevalence of anxiety (4), depression (5), PTSD (6), and sleep disturbances (7). Further research indicates that people may be reluctant to adhere to the social distancing restrictions because of the perceived impact on their mental health (8). Even though social distancing restrictions and uncertainty affect the whole population, it seems evident that a significant part of the population has been living through the pandemic without a severe mental or physical impact. This raises a question about how the remaining population has been coping, where a focus on psychological well-being can add to a more comprehensive understanding of the overall psychological status.

Recognizing mental health as more than the absence of illness, World Health Organization (WHO) declared well-being as a fundamental aspect of positive mental health, which can have profound consequences by facilitating effective functioning in everyday life (9). Well-being covers a broad range of aspects of life, such as subjective well-being, psychological and social functioning, and professional life. Individuals experiencing high levels of well-being may cope better with stress, realize their abilities and goals, and contribute to society (10).

Two significant perspectives, hedonic and eudaimonic well-being, have been adopted in research on well-being. Hedonic well-being has emphasized well-being as a subjective experience of maximizing happiness and pleasure (11). For the eudaimonic perspective, well-being is experiencing meaning, involvement, and self-realization through psychological well-function and human growth. This approach suggests that subjective happiness is distinct from well-being (11) and emphasizes that negative feelings and experiences are integral to human life and growth (12). The experience of meaning and purpose (13) and participating in social engagement (14) have been listed as particularly principal in the eudaimonic approach. The COVID-19 pandemic has required extensive restrictions in social, educational, and professional life (2). These restrictions challenge aspects of both hedonic and eudaimonic well-being. The uncertainty of the pandemic duration, combined with the consequences of the political restrictions, has led to a more unstable economy and labor market (15). Many people have lost their job temporarily or permanently (15), and the future employment market is uncertain. This uncertainty may be reflected in the individual's experience of being useful, experiencing less happiness, or feeling less optimistic about the future. The unpredictable circumstances may increase personal stress and a reduced ability to think clearly and solve problems. Social distancing is further a potential risk factor for feeling less close and connected to other people. It seems evident that the pandemic is a potential threat to mental well-being among the general population.

Under the challenging but unavoidable pandemic circumstances, it is essential to investigate factors that contribute to changes in well-being, as such investigations could provide a preliminary basis of factors relevant for increasing individual well-being. These factors may include context-specific factors and personal psychological processes and behaviors. Concerning maladaptive psychological processes, the self-regulatory executive function model (S-REF) proposes that a syndrome of thinking styles called cognitive attentional syndrome [CAS; (16)] has adverse effects on psychological functioning. CAS is characterized by excessive conceptual processing, such as worrying, rumination, attentional focus on threats, and unhelpful coping behaviors. CAS results from activating two broad styles of metacognitive beliefs: positive and negative metacognitions. Metacognition is defined as the control, modification, and interpretation of thoughts and feelings (17), i.e., how people think about their thoughts, feelings, and the perceived control they might have over them. Positive metacognitive belief is characterized by engaging in cognitive activities that constitute the CAS, emphasizing the perceived positive consequences and usefulness of focusing on threats and worry (16). Negative metacognitions concern the uncontrollability, importance, and dangerousness of thought and cognitive experiences. Both positive and negative metacognitions are theorized to have an adverse effect on psychological functioning (16). Given the strict social distancing restrictions and uncertainty regarding the future of the pandemic, positive and metacognitions may lead to increased worrying, rumination, and coping mechanisms, which can intensify and prolong a negative emotional experience. Furthermore, this may be associated with reduced optimism, withdrawal from everyday life, and feeling less close to other people, which all are aspects of reduced well-being. Threat monitoring takes up attentional resources (16), impairing the ability to think clearly and deal with problems appropriately. These negative thinking patterns and unhelpful coping strategies may further increase individual stress. Thus, the processes and metacognitive beliefs in the CAS may adversely affect both hedonic and eudaimonic well-being.

It is highlighted that CAS contributes to and maintains different psychological disorders, such as generalized anxiety disorder and depression (16, 18). As both anxiety and depression are related to lower well-being (19), it is essential to control these potential confounders when studying the relationship between CAS and well-being.

Being physically active is a fundamental factor in predicting better mental health and well-being. A meta-analytic review shows that being physically active positively affects subjective well-being across all age groups (20). Studies have further demonstrated that physical activity can be used to improve the quality of life and mental health (21). For instance, being physically active may improve mental well-being in public (22).

Employment status is another well-known predictor of well-being. Being unemployed negatively affects mental well-being (23), where unemployed people report lower well-being than employed individuals (24). Being unemployed may generate economic distress and decreased control over the future, which profoundly can harm individual well-being. Being employed further contributes to essential psychosocial functions for individuals. Depending on the cultural and societal context, work can be an essential part of social identity and the opportunity to partake in society in a meaningful way. Being employed provides a time structure, regular activity, and social contact (23). In the month following the lockdown in Norway, the unemployment rates rose from 2.3 % in February 2020 to 10.7 % in March 2020 (15). Comparing with March of 2019, the unemployment rate has more than tripled in March 2020 (15). Losing one's job due to the social distancing restrictions was a more prevalent outcome amongst younger individuals' [aged 39 and below; (15)].

The aim of this study was to investigate the level of well-being after the partial lifting of strict social distancing restrictions (T2), and investigate factors associated with well-being in the general population. The additional predictive effect of variables measured at a period of strict social distancing restrictions (T1) were also investigated. The following research question was: What is the level of well-being following 3 months of strict social distancing restrictions (i.e., physical distancing) in the general adult population during the COVID-19 pandemic? The mean level of mental well-being will be benchmarked against the mean level of mental well-being in similar pre-pandemic samples. Furthermore, several factors assessed at T1 and T2 were investigated to reveal their associations with well-being, giving rise to the following hypothesis:

### Hypothesis 1

Higher levels of positive metacognitions, negative metacognitions, and unhelpful coping strategies at T2 will be significantly associated with lower well-being. This hypothesis investigates the contemporaneous association between these variables and well-being.

### Hypothesis 2

Being employed and physically active at T2 is associated with higher concurrent well-being.

### Hypothesis 3

Higher levels of positive metacognitions, negative metacognitions, and unhelpful coping strategies at T1, indicating the previous levels and amounts of the variables, will further contribute to the levels of well-being, over and above the influence of the concurrent levels at T2.

In investigating these three hypotheses, depressive symptoms (PHQ-9) and anxiety symptoms (GAD-9) at T2 and T1 will be included to control for these variables' as potentially mediating or confounding factors when studying the relationship between well-being and hypothesized predictors of well-being.

## Methods

### Study design and participants

This study is part of The Norwegian COVID-19, Mental Health and Adherence Project. The design is an observational survey of the general adult Norwegian population. Participants were 18 years of age and above who were residing in Norway and consequently experiencing identical social distancing restrictions. Ethical approval of the study was granted by the Regional Committee for Medical and Health Research Ethics and the Norwegian Center for Research Data (reference numbers: 125,510 and 802,810, respectively). The participants provided written informed consent to participate in the study.

Data were collected during two separate time intervals. The first data collection was between March 31st, 2020, until April 7th, 2020 (T1). The social distancing restrictions were implemented from March 12th, 2020, and kept constant for 2 weeks prior to and during the entire week of the first data collection. There were no new information or changes regarding the social distancing restrictions during the data collection, thus controlling for expectation effects. A total of 10 061 people completed the first survey. On June 15th, multiple of the strict distancing restrictions were lifted. The second data collection was gathered from the previous sampling, and lasted for 3 weeks, from June 22nd, 2020, until July 13th (T2), where 4,921 (49 %) of the original sample responded. Well-being was assessed only at T2. Supplementary Tables S1,S2 reveals the differences and changes in the social distancing restrictions in-practice during the first wave of data collection (T1) and the second wave of data collection (T2).

### Procedures

An online survey was distributed to Norway's adult population in a systematic process to maximize equal opportunity to participate in the study and obtain a probability sample. The survey was predominantly disseminated to a random selection of Norwegian adults on Facebook, targeted through a Facebook Business algorithm designed to provide each adult on Facebook with an equal probability of receiving the survey. Approximately 85% of the Norwegian adult population are available on Facebook, thus indicating that 15% of the adult population were not reachable with this algorithm. To maximize the probability of reaching out to the latter 15% of the adult population, the survey was also distributed through broadcasting on national, regional, and local news channels, radio stations, and newspapers. Only one of these six platforms (national news channel) had more than 1.1 million viewers at the time of broadcast. Further details about this process may be found here (25).

### Measures

Participants reported various demographic variables, such as age, sex (male, female), educational status (no higher education, finished a university degree and currently undertaking university degree), and employment status (yes, no). Physical activity was measured as the number of times being active over 30 min with moderate activity within the last 2 weeks (not at all, one time, 2–3 times, 4–8 times, more than eight times).

Participants reported the number of days out of the last 14 days, where they had followed the governmental restrictions of keeping a social distance. Individuals reporting to have been socially distanced for at least 10 of the last 14 days were coded as predominantly socially distanced. Supplementary Tables S1,S2 includes the different socially distancing restrictions in practice during the first (T1) and second (T2) waves of data collection.

#### The short Warwick-Edinburgh mental well-being scale

The Short Warwick-Edinburgh Mental Well-being Scale (SWEMWBS) consists of seven items related to subjective well-being and psychological functioning. The scale covers positive feelings and thoughts (e.g., I have been feeling optimistic about the future) and coping [e.g., I have been dealing with problems well; (26)]. Thus, attributes of hedonic and eudaimonic well-being are covered in the scale. However, there is not established which items belong to each of the two distinct perspectives. The items are scored on a five-point Likert scale, and a composite score was computed by summing the items after reverse coding, with composites ranging from 7 to 35. A higher score on the items reflects higher subjective well-being. Cut-off indicting low well-being was set to a score of 19 or below (27). Psychometric properties are acceptable in the Norwegian translation of the scale (28), and the internal consistency in the present sample was good, with Cronbach's α of 0.877.

#### Cognitive attentional syndrome-1 scale

Scores of positive and negative metacognitions, including unhelpful coping strategies, were measured by the Cognitive Attentional Syndrome-1 scale (CAS-1). The scale includes items concerning worrying, rumination, and maladaptive coping behavior related to negative thoughts and emotions, such as ‘how much time in the last week have you found yourself dwelling on or worrying about your problems?’. The scale further includes items connected to positive and negative metacognitions, such as ‘worrying helps me cope’ or ‘worrying too much could harm me’ (29). Internal consistency was excellent in the current sample with Cronbach's α of 0.904.

#### Patient health questionnaire-9

Depression was assessed through the Patient Health Questionnaire-9 (PHQ-9), which consists of nine items that covers the DSM-IV criteria for major depression. Items are scored on a four-point Likert scale, where total scores range from 0 to 27. Higher scores indicate greater depression severity (30). Internal consistency in the present sample was good with a Cronbach's α of 0.884.

#### Generalized anxiety disorder-7

Measurements of general anxiety were assembled by the Generalized Anxiety Disorder-7 (GAD-7) scale, which contains seven items covering the DSM-IV criteria for GAD. The items are scored on a four-point Likert scale, where total scores are ranging from 0 to 21; higher scores indicate more severe anxiety severity (31). Internal consistency in the sample was good, with Cronbach's α of.880.

### Statistical analysis

A hierarchical regression analysis was conducted, with a composite score of the SWEMWBS-variables as the dependent variable. The statistical analysis was conducted in R (version 4.0.2). In the first step, stable characteristics as age, sex, and education were included. Depressive and anxiety symptoms at T2 were added as control variables. In the second step, physical activity, being employed, being socially distanced, positive metacognitions, negative metacognitions, and unhelpful coping strategies at T2 were added. In the third step, physical activity, being employed, positive metacognitions, negative metacognitions, and unhelpful coping strategies at T1 were included in the analysis. This step further included depressive and anxiety symptoms at T1 as control variables. Moreover, social distancing was added to the regression in this step to investigate the unique relationship between social distancing and well-being after controlling for all aforementioned variables. Consequently, the analysis involved strictly controlled predictions of well-being regarding possible confounding variables, controlling for multiple relevant variables in addition to concurrent and prior levels of psychopathological symptoms.

Given the large sample size, the predefined significance levels were set to *p* < 0.01. Multicollinearity and other statistical assumptions were assessed with standard guidelines, given VIF < 5 (32). Part correlations are provided during each step to present the hypothesized predictors' effect size in its association with well-being.

### Sensitivity analysis and weighting

Given that participation in the present study was voluntary, the study was susceptible to over- and undersampling of specific subgroups of participants, and thus to some extent, deviation from the accurate population distribution of these subgroups. To deal with this concern and subsequently apply the most accurate and conservative approach concerning inference to the general Norwegian adult population, appropriate weights were assigned to match the over- and undersampled subgroups to their precise population distributions. In this study, these subgroups included sex, age, ethnicity, education, and geographic region. This procedure assigns each overrepresented and underrepresented group weights proportionate to their distribution and frequency in the population. More specifically, more weight is assigned to underrepresented groups, and less weight is put on overrepresented groups, resulting in a highly representative sample of the Norwegian adult population. The weighting procedure was conducted utilizing the R-packages ‘anesrake’ and ‘survey’. An iterative algorithm (i.e., raking ration estimation) was used to avoid that the matching of the distribution of one variable unmatches the distribution of another. This iterative algorithm weights variables by turn, leading to a converging set of weights for each factor that closely matches subgroups to their population distribution.

## Results

### Characteristics of participants

A total of 10 061 responded in the first data collection at T1. Of these individuals, 4,936 (49.10%) responded to the second data collection (T2). In T2, the age distribution ranged from 18 to 86 years, with a mean age of 39 years. Moreover, 79.20% (*n* = 3911) of the respondents were females, and 20.50 % (*n* = 1,010) were males. A total of 4,921 participants were employed in the hierarchical regression model, as two levels of the sex variable (i.e., intersex and transgender) contained too few participants (*n* = 15) to be included as separate factors in the regression analysis. After the weighting procedure, the sample reflected a more precise distribution of the Norwegian population and sub-groups. A complete overview of the sampled and weighted population is presented in Table 1.

**Table 1:** The proportion of the sample participants.

	**Sampled *N* (%)**	**Weighted *N* (%)**	**Percentage of subgroup in the Norwegian adult population**
**Sex**			
Female	3,911 (79.48%)	2,585 (52.53%)	49.77%
Male	1,010 (20.52%)	2,336 (47.47%)	50.23%
**Age group**			
18–30	1,703 (34.07%)	1,245 (25.30%)	23.20%
31–44	1,606 (32.64%)	1,242 (25.24%)	24.30%
45–64	1,344 (27.31%)	1,630 (33.12%)	31.26%
65 and above	268 (5.45%)	804 (16.34%)	21.22%
**Ethnic background**			
Native	4,563 (92.27%)	4,408 (89.56%)	85.29%
Europe	274 (5.57%)	343 (6.97%)	7.58%
Asia	39 (0.79%)	117 (2.38%)	4.56%
Africa	6 (0.12%)	18 (0.37%)	1.85%
North America and Oceania	15 (0.30%)	14 (0.28%)	0.27%
Middle- and South America	24 (0.49%)	21 (0.43%)	0.45%
**Region**			
East Norway	3,103 (63.06%)	2,943 (59.81%)	58.32%
West Norway	1,162 (23.61%)	893 (18.14%)	20.28%
Mid-Norway	482 (3.54%)	833 (16.92%)	15.95%
Northern Norway	174 (3.54%)	252 (5.13%)	5.45%
**University degree**			
Yes	3,219 (65.41%)	1,835 (37.29%)	30.09%
No	1,702 (34.59%)	3,086 (62.71%)	69.91%

A table revealing the proportion of the sampled participants. All oversampled and undersampled subgroups were assigned appropriate weights to reflect their known distribution in the population as precisely as possible. The raking ratio algorithm converged with the adjustments weighting the sex, age, ethnic background, and regional location of the participants.

### Level of well-being

Alle the subsequent results are based on the weighted sample, and results of well-being among different subgroups are presented in Table 2. The mean score of well-being was 25.38, indicating moderate well-being in the population. The number of individuals scoring at cut-off or below, indicating low well-being, was 675 (13.72 %). More females than males scored below the cut-off, and results also show that the younger population was more likely to score below the cut-off. Furthermore, more unemployed individuals scored below the cut-off compared to those employed. Individuals who did not have higher education were also more likely to score below the cut-off for low well-being.

**Table 2:** Table of scores of well-being (SWEMWBS).

**Subgroup**	***M (SD)***	***N* (%)**	**Below cut-off^a^ (%)**
**All participants**	25.38 (5.24)	4,921 (100%)	675 (13.72%)
**Sex**			
Male	26.08 (5.08)	2,336 (47.47%)	236 (10.10%)
Female	24.75 (5.31)	2,585 (52.53%)	439 (16.98%)
**Age group, years**			
18–30	23.80 (5.03)	1,245 (25.30%)	237 (19.04%)
31–44	24.65 (5.41)	1,242 (25.24%)	217 (17.47%)
45–64	26.12 (5.19)	1,630 (33.12%)	184 (11.29%)
65+	27.47 (4.38)	804 (16.34%)	36 (4.48%)
**Higher educated**			
Not higher educated	24.61 (5.35)	3,086 (62.71%)	549 (17.79%)
Finished university or college degree	26.68 (4.78)	1,835 (37.29%)	126 (6.87%)
**Employment status**			
Current employed	26.14 (4.60)	3,088 (62.75%)	249 (8.06%)
Current unemployed	24.10 (5.96)	1,833 (37.25%)	426 (23.24%)

a The cut-off value for the SWEMBS was defined as 19.

There was a significant difference (*t* = 8.20, *p* < 0.001, *d* =0.27) in concurrent well-being between those who predominantly socially distanced themselves at T2 (M = 25.03, SD = 5.27) as compared to those who did not predominantly socially distance themselves (M = 26.43, SD = 5.01). The difference between these two groups was small to moderate (*d* =0.27), revealing that those who were concurrently predominantly socially distanced were associated with lower well-being levels. Additionally, there was a significant temporal association (*t* = 6.03, *p* < 0.001, *d* =0.21) between social distancing at the early stages of the pandemic (i.e., T1) and current levels of well-being (T2). Once again, those who predominantly socially distanced themselves reported lower well-being (M = 25.14, SD = 5.33) than their counterparts (M = 26.22, SD = 4.82). This relationship was further investigated to inspect whether any direct association remained between social distancing and well-being when controlling for other related variables, reported below in the sensitivity analysis section.

### Predictors of well-being

The results of hierarchical regression analyses for well-being as the dependent variable are presented in Table 3. Results show that age, sex, and education levels were unrelated to well-being when controlling for concurrent psychopathological symptoms (i.e., anxiety and depressive symptoms at T2) in a weighted sample. Results further indicate that both depressive (part correlation = −0.250) and anxiety symptoms (part correlation = −0.115) at T2 is significantly related to lower well-being. Variables in step one explained 62 % of the variance in well-being, adjusted R^2^ = 0.622.

**Table 3:** Results of hierarchical regression with well-being (SWEMWBS) as the dependent variable.

**Variables**	***B***	***SE***	***t***	***p***	**Part correlations**	***R* ^2^**	**ΔR^2^**
**Step 1 Stable characteristics**						**0.622**	**0.622**
Age	<0.001	0.005	0.103	0.918	*0.002*		
Sex	−0.263	0.164	−1.602	0.109	*−0.037*		
Education	0.029	0.176	0.164	0.870	*0.003*		
T2 Anxiety symptoms	−0.276	0.032	−8.622	<0.001	*−0.115*		
T2 Depressive symptoms	−0.499	0.025	−19.772	<0.001	*−0.250*		
**Step 2 Psychosocial variables at T2**						0.644	0.022
T2 Physical activity	0.130	0.064	2.023	0.043	*0.049*		
T2 Employed	0.503	0.184	2.732	0.006	*0.057*		
T2 Positive metacognitions	0.004	0.001	3.302	<0.001	*0.072*		
T2 Negative metacognitions	−0.005	0.001	−3.963	<0.001	*−0.078*		
T2 Unhelpful coping strategies	−0.087	0.014	−6.295	<0.001	*−0.089*		
T2 Socially distanced	−0.216	0.171	−1.269	0.204	*−0.029*		
**Step 3 Psychosocial variables at T1**						0.651	0.007
T1 Physical activity	0.007	0.071	0.103	0.918	*0.004*		
T1 Employed	−0.469	0.282	−1.661	0.097	*−0.024*		
T1 Positive metacognitions	0.004	0.001	3.500	<0.001	*0.059*		
T1 Negative metacognitions	−0.006	0.001	−4.220	<0.001	*−0.064*		
T1 Unhelpful coping strategies	−0.008	0.013	−0.652	0.514	*−0.008*		
T1 Depressive symptoms	−0.042	0.028	−1.498	0.134	*−0.017*		
T1 Anxiety symptoms	0.036	0.036	0.992	0.321	*0.012*		
T1 Socially distanced	−0.060	0.181	−0.333	0.739	*−0.016*		

SWEMWBS, The Short Warwick-Edinburgh Mental Well-being Scale.

In the second step, the regression model accounted for 64 % of the variance in well-being, adjusted R^2^ = 0.644. Concurrent employment status (i.e., T2) was related to better well-being (part correlation = 0.057). Physical activity was not related to concurrent well-being. Negative metacognitions (part correlation = −0.078) and unhelpful coping strategies (part correlation = −0.089) at T2 were related to reduced well-being. Positive metacognitions (part correlation = 0.072) at T2 were related to higher scores of well-being.

With regards to prior psychosocial variables at the initial stages of the pandemic (i.e., T1), both negative metacognitions (part correlation = −0.063) and positive metacognitions (part correlation = 0.059) at T1 were associated with well-being, even after strictly controlling for the influence of the current levels of the psychosocial variables as well as the influence of depression and anxiety symptoms at both time-points. Prior levels of negative metacognitions were negatively related to well-being, indicating that higher levels of metacognitions at T1 are associated with lower well-being scores. Positive metacognitions were, on the other hand, positively related to well-being. Being physically active, employed, and using unhelpful coping strategies at T1 were not related to well-being. In total, including the three steps, the model explained 65 % of the variance in well-being, with adjusted R^2^ = 0.651.

### Sensitivity analysis inspecting the relationship between social distancing and well-being

Results from the multiple regression (Table 3) showed that being predominantly socially distanced was not statistically significantly related to well-being when controlling for all other 18 variables in the model. This is in contrast with the initial analysis investigating the bivariate association between being socially distanced showing an association with lower well-being. This may indicate that the association between social distancing and well-being is an indirect one going through one of the other investigated variables. Accordingly, we inspected the sensitivity of the results through a mediation analysis (33) investigating whether depression and anxiety, shown in the multiple regression as two of the most significant contributors to changes in well-being, mediated the relationship between being socially distanced and well-being. The mediation analysis was conducted in R (version 4.0.2). Given the criticisms of conducting mediation analysis with all variables on the same time-point [i.e., without any temporal precedence; (34)], we conducted the mediation analysis investigating the temporal association between social distancing at T1 on depression, anxiety and well-being at T2.

The indirect relationship of social distancing through depression was significant (estimate = −1.25, *p* < 0.01), revealing lower well-being following an associated heightening in depression. The strength of evidence for the indirect relationship of social distancing through anxiety was not equally strong and insignificant concerning our studies' pre-specified alpha level (estimate = −2.02, *p* = 0.024), though revealing similar patterns of lower well-being following an associated increase in anxiety. After accounting for these indirect effects, no significant direct association remained between social distancing and well-being (estimate = −0.70, *p* = 0.126).

## Discussion

### Level of well-being

Due to the lack of data on the studied sample before the pandemic, we cannot evaluate whether the level of well-being was lower than during non-pandemic times. With regards to the limited literature on well-being during epidemic or pandemic circumstances, a comparison with results of well-being in European non-pandemic populations is the closest benchmark. Findings from England in 2010–2013 using SWEMWBS found that women's level was 23.6 and 23.7 for men [*n* = 27,169; (35)] and other results from the UK identified that well-being among adults (16 years and older) was 25.0 [*n* = 38,395; (24)]. Overall results of mean well-being at SWEMWBS in Nordic countries in non-pandemic samples show 25.4 in Iceland in 2017 and 26.4 in Denmark in 2016 (36). Further studies conducted of Norwegian adolescents support the same pattern, scoring a mean result of well-being at 24.9 (*n* = 1,679), measured by the SWEMWBS-scale (37). Compared with non-pandemic samples in other European countries, it is reasonable to conclude that Norway's adult population's general well-being was not remarkably reduced during the pandemic. A counterargument is that the Norwegian population traditionally has been ranked as one of the happiest countries from 2018 until 2020 (38–40). Consequently, one could conclude that the general level of well-being in Norway should be higher than in the benchmarked countries. However, both Denmark and Iceland score higher than Norway in the happiness ranking in these years (38–40) which further complicates this understanding.

It is debatable whether more individuals score below the cut-off for low well-being due to COVID-19 and the profound consequences in everyday life. Studies from the UK in a non-pandemic population have used 19 as a cut-off score to indicate low well-being, defining approximately 15% of the group participants to score below the cut-off (24). Our results show that only 13.4 % of the respondents scored at cut-off or lower, which could indicate that the group scoring at impaired well-being might not have increased during the pandemic and after implementing social distancing interventions, compared to a non-pandemic population. However, a published meta-analysis has inferred that the prevalence of stress, anxiety, and depression has increased during the COVID-19 pandemic (41). These results are supported by findings from Norway, indicating that the prevalence of loneliness (42), anxiety, and depression (25) has increased at the same time. A critical question is whether the group scoring at low well-being may suffer even worse well-being than earlier, in the light of the strict social distancing restrictions in Norway.

### Anxiety, depression, physical activity and employment status

Results indicate that 62 % of the variance of well-being is due to the variables in step one, where anxiety and depression were the only significant variables. Our results show that higher scores for depression and anxiety are associated with lower well-being. It seems evident that anxiety and depression are two of the most significant contributors to changes in well-being.

It was further hypothesized that physical activity would be positively related to well-being in light of pre-pandemic research (20–22) and research conducted during the pandemic and lockdowns (43, 44). Given that there was no significant connection between physical activity at T2 and well-being, it might be due to the inclusion of scores of anxiety and depression. Accordingly, it may be that previous beneficial associations between physical activity and well-being are indirect through reduction of symptom of depression and anxiety, with the latter association between physical activity and reduction in these symptom domains previously identified in the literature (45, 46).

There is support for contemporaneous employment at T2 being related to better well-being. Individuals not employed at T2 are more prevalent in the group scoring below the cut-off for low well-being, further supporting the significance of employment status and well-being. Non-pandemic samples of full-time employees report higher well-being than unemployed, part-time employees, or long-term sick. Low well-being is more common among unemployed men, but the results may be influenced by other factors, such as mental ill-health [e.g., depression and anxiety; (24)]. Our results indicate that the current employment status (i.e., T2) is associated with well-being.

### Positive and negative metacognitions and unhelpful coping strategies

Results show that negative metacognitions are associated with reduced well-being both contemporaneous and across time, even when controlled for anxiety and depressive symptoms. Furthermore, this indicates that non-adaptive thinking styles are associated with reduced well-being in the general population and not exclusively a clinical sample. This indicates that CAS is directly associated with lower well-being in individuals not plagued with depressive and anxiety symptoms. It is stated that exposure to stressful life events increases engagement in rumination longitudinally (47), which may serve as a risk factor for decreased well-being and life quality.

Positive metacognitions are traditionally related to reduced psychological functioning and increased psychopathology (16), considering positive metacognitions lead to more engaging in unhelpful coping strategies and maladaptive thinking-styles (48). In contrast to the traditional theoretical understanding, our results show that positive metacognitions were positively related to better well-being. One possible reason for this finding may be an artifact of measurement or related to the measurement context. Specifically, some items measuring metacognition in the CAS-1 instrument concern contemporary (i.e., past-week) beliefs about how 'focusing on possible threats make me safe' (29). While such a focus in non-pandemic settings may be related to hyper monitoring, a mechanism generally associated with more psychological stress, it is possible that such cognitions function differently in the pandemic contexts, especially during the early stages of the pandemic where substantial portions of information were unknown about the virus (e.g., the different ways through which it can transmit; its dangerousness; its long-term consequences). As such, it may be possible that such a cognition was facilitative for well-being in the specified pandemic context, explaining the positive association between positive metacognitions and well-being in this sample.

### Social distancing and well-being

Studies have identified detrimental associations between social distancing and depression through the pandemic (49, 50), and anxiety (51, 52) during the onset of the pandemic. Consistent with the literature, the conducted mediation analysis revealed that the temporal association between social distancing and lower well-being was indirect and mediated through heightened states of depression. When these variables were controlled for, there remained no significant direct association between social distancing and well-being. The strength of evidence was more robust for the indirect pathway through depression, meeting the pre-specified significance criteria (*p* < 0.01), than for anxiety (*p* = 0.024). These results are consistent with previous studies identifying links between social distancing protocols and depression, but not for anxiety, which was more strongly related to infection rates (49). This finding is an important extension to the literature in identifying the pathways through which well-being may have been impacted during the pandemic.

Notably, engaging in cognitive focus on threats was related to increased well-being, while findings showed that addressing such threats through behavior by social distancing was associated with reduced well-being, mediated through heightened depressive symptoms. As previously discussed, a cognitive focus on possible threats during the pandemic (e.g., getting infected, infecting others) could serve as a calming mechanism in a novel and unpredictable situation. However, different associations were found between engaging in this cognitive activity vs. engaging in behavior related to the virus, the latter which mainly allowed for the strategy of social distancing (1, 2), including isolation from family and friends, loss of social contact, and decreased participation pleasurable activities (1). As such, while a cognitive focus on possible threats in a novel and for many unprecedented situations may have been favorable for well-being, it seems a possible byproduct of the behavioral change accompanying this was related to lowered well-being, which may be related to increased rates of loneliness (53), further linked to increases in depressive symptoms (54).

### Strengths and limitations

The sensitivity analysis and weighted dataset closely matched the Norwegian population parameter, strengthening the results' generalizability. Considering the number of variables included in the regression analysis and the strict, predetermined significance level further strengthens the robustness of our results. The larger sample obtained in this study further contributes to increased statistical power.

A significant limitation of this study is the lack of pre-pandemic data on the general well-being of the sample and the Norwegian population. Thus, is it challenging to determine a change in well-being and eventual increase of individuals scoring below the cut-off. It is further not possible to conclude causal effects since the methodological design does not meet the strict criteria of causality. Voluntary participation in the study may also contribute to a bias in the sample, where sensitivity analysis was conducted to reduce the influence of these potential effects. The last limitation is the lack of standardized instruments in assessing physical activity and being socially distanced, which may bias the measurements.

## Conclusions

Results from this study show that negative metacognitions and the use of unhelpful coping strategies were associated with lower well-being. Conversely, employment status and positive metacognitions were positively related to well-being, either contemporaneous and/or across time. These results cast light on significant predictors for changes in well-being after the lifting of strict social distancing restrictions. Adherence to governmental restrictions was an essential factor for reducing virus transmission, but there seems to have been some psychological cost to this. With regards to well-being specifically, these costs seem to have been indirect through heightened states of depressive symptoms. The findings from this study contribute to the understanding of possible psychological biproducts after the implementation of social distancing restrictions, which may further contribute to the design of proper strategies and programs aiming at strengthening mental health and well-being during challenging societal conditions.

## Data availability statement

The data analyzed in this study was obtained from Omid V. Ebrahimi and Sverre Urnes Johnson. The ethical approval granted for this study precludes the data from being published at a public respiratory, following ethical approval of a suggested project plan for the use of data granted by NSD and REK. Requests to access these datasets should be directed to Omid V. Ebrahimi, omideb@uib.no.

## Ethics statement

Ethical approval of the study was granted by the Regional Committee for Medical and Health Research Ethics and the Norwegian Centre for Research Data (reference numbers: 125510 and 802810, respectively). The patients/participants provided their written informed consent to participate in this study.

## Author contributions

SE performed part of the data analysis, interpreted the results, and wrote the manuscript. OVE, SUJ, and AH designed the study, collected the data, and revised the manuscript. NS performed the data analysis and wrote part of the manuscript. All authors contributed to the article and approved the submitted version.

## Conflict of interest

The authors declare that the research was conducted in the absence of any commercial or financial relationships that could be construed as a potential conflict of interest.

## Publisher's note

All claims expressed in this article are solely those of the authors and do not necessarily represent those of their affiliated organizations, or those of the publisher, the editors and the reviewers. Any product that may be evaluated in this article, or claim that may be made by its manufacturer, is not guaranteed or endorsed by the publisher.
